# Immobilization of Polymeric Luminophor on Nanoparticles Surface

**DOI:** 10.1186/s11671-016-1410-0

**Published:** 2016-04-18

**Authors:** Yuliia Bolbukh, Beata Podkoscielna, Agnieszka Lipke, Andrzej Bartnicki, Barbara Gawdzik, Valentin Tertykh

**Affiliations:** Chuiko Institute of Surface Chemistry of National Academy of Sciences of Ukraine, 17 General Naumov Str., 03164 Kyiv, Ukraine; Faculty of Chemistry, Maria Curie-Skłodowska University, pl. Maria Curie-Skłodowskiej 3, 2-031 Lublin, Poland

**Keywords:** Multiwalled carbon nanotubes, Silica nanoparticles, Polymeric luminophor, Chemisorption, Luminescence properties, 66.30.hk, 63.22.Gh, 78.20.Ek

## Abstract

Polymeric luminophors with reduced toxicity are of the priorities in the production of lighting devices, sensors, detectors, bioassays or diagnostic systems. The aim of this study was to develop a method of immobilization of the new luminophor on a surface of nanoparticles and investigation of the structure of the grafted layer. Monomer 2,7-(2-hydroxy-3-methacryloyloxypropoxy)naphthalene (2,7-NAF.DM) with luminophoric properties was immobilized on silica and carbon nanotubes in two ways: mechanical mixing with previously obtained polymer and by in situ oligomerization with chemisorption after carrier’s modification with vinyl groups. The attached polymeric (or oligomeric) surface layer was studied using thermal and spectral techniques. Obtained results confirm the chemisorption of luminophor on the nanotubes and silica nanoparticles at the elaborated synthesis techniques. The microstructure of 2,7-NAF.DM molecules after chemisorption was found to be not changed. The elaborated modification approach allows one to obtain nanoparticles uniformly covered with polymeric luminophor.

## Background

Materials with luminophor properties are widely used in the production of lighting devices, sensors, detectors, bioassays or diagnostic systems. The most ambitious and fascinating application of luminescent nanomaterials is probably related to medicine and molecular biology. Luminescent nanomaterials are promising tags for optical imaging and fluorescent labelling to allow for novel techniques of non-invasive diagnosis and in vivo observation of complex vital functions [1]. The recent advances in nanotechnology have demanded to raise a new class of fluorescent labels. Researchers have developed several different types of luminescent nanomaterials, including semiconductor quantum dots, dye-doped nanoparticles, up-converting lanthanide-doped nanoparticles and carbon nanodots on silica surface [2]. From this point of view, the optical properties of carbon nanotubes have attracted much recent attention. For example, there have been extensive investigations of carbon nanotubes as optical limiters for attenuating pulsed laser irradiation [3]. Apparently, the functionalization of the nanotubes resulted in more intense luminescence emissions [4]. Along with existing nanoscale materials [5, 6], modified nanotubes with luminescence properties have potential as materials for bioanalytical assays and luminescent imaging [7]. That is why the development of composites based on polymeric luminophor and nanoparticles attracted a considerable interest. One of the priorities is the development of polymer luminophors with reduced toxicity. Such polymers can extend the luminophor applications in biotechnology and medicine, including the creation of biomarkers. Recent advances in surface grafting of polymers onto carbon materials, such as carbon black, graphite powder and carbon nanotubes, and the application of polymer-grafted carbon materials as novel functional materials have been mainly reviewed [8–10]. The grafting of polymers onto the surface can be achieved by (1) *grafting onto* process [11], (2) *grafting from* process [12], (3) *polymer reaction* process [13] and (4) *stepwise growth by dendrimer* synthesis methodology [14]. For polymer anchorage, the surface functional groups, such as carboxyl and phenolic hydroxy groups, were used as grafting sites [15]. For example, in the living cationic polymerization initiated by carbon black/ZnCl_2_ system, carboxyl groups on the surface acted as initiating and grafting sites. The in situ atom transfer radical polymerization (ATRP) *grafting from* approach was successfully applied to graft poly(methyl methacrylate) (PMMA) onto the convex surface of multiwalled carbon nanotubes (MWCNTs) [16]. The thickness of the coated polymer layers can be conveniently controlled by the feed ratio of MMA to preliminarily functionalized MWCNT (MWCNT-Br). Moreover, the approach has been extended to the copolymerization system, affording novel hybrid core–shell nano-objects with MWCNTs as the core and amphiphilic poly(methyl methacrylate)-block-poly(hydroxyethylmethacrylate) (PMMA-b-PHEMA) as a shell.

Earlier, we elaborated the approach [17] that allows to obtain carbon nanotube-based nanomaterials with tailor-made structure and properties. The MWCNTs were modified with monomer 2-hydroxyethylmethacrylate using radical process or polycondensation reaction to obtain different functional groups, namely hydrophobic (methacrylic) or hydrophilic (hydroxyl), in the boundary layer. Along with silica surface functionalization, the modification of MWCNTs with functional silanes was evaluated and optimized based on accumulated experience [18, 19].

### Background of the Study

For polymeric luminophors, the fluorescent light mostly appears in a thin surface layer without participation of the polymer in volume. Obtaining of the polymeric surface layer on a surface of nanoscale particles while maintaining its luminescent properties is of considerable practical interest. The high specific surface of nanoparticles supposed should promote better contact of luminophor with light radiation, and thus, luminescence should increase at lesser amounts of polymer. Moreover, utilization of the nanoparticles with immobilized luminophor has to provide target localization light-sensitive markers. The aim of this study was to develop a method of immobilization of the monomeric luminophor on a surface of nanoparticles based on elaborated approaches and investigation of the structure of the grafted layer in the modified products.

## Methods

The monomer 2,7-(2-hydroxy-3-methacryloyloxypropoxy)naphthalene (2,7-NAF.DM) was used for immobilization [20]. The oxidized H_2_O_2_ [17] multiwalled nanotubes produced in the Chuiko Institute of Surface Chemistry (Ukraine) using the catalytic vapour deposition method [21] and pyrogenic silica with a specific surface area of 260 m^2^/g (A-300, Kalush, Ukraine) were utilized as carriers. In order to improve monomer–nanoparticle interaction, nanotubes and silica were activated via attaching of the vinyltriethoxysilane (Fluka) that was used without addition treatment. Analytical grade solvents were purchased from Sigma-Aldrich.

### Synthesis of Monomer 2,7-NAF.DM

**Scheme 1:**
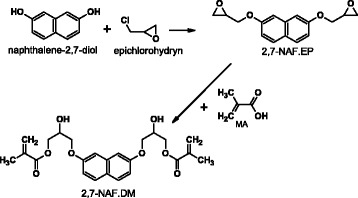
Synthesis of 2,7-NAF.DM

The 2,7-NAF.DM was obtained in the two-step reaction. In the first step, naphthalene-2,7-diol (2,7.NAF) was reacted with epichlorohydrin (EP) in the two-phase liquid/liquid system including organic and aqueous phases. Next, esterification of the obtained diglycidyl ether was carried out using methacrylic acid (MA) in the presence of hydroquinone (polymerization inhibitor) and triethylbenzylammonium chloride as a catalyst. Finally, the aromatic monomer (2,7-NAF.DM) with photoluminescent properties was prepared (Scheme 1). The detailed information about synthesis was presented elsewhere [20, 22].

With the use of a spectrofluorimeter, the emission and excitation spectra were measured for 2,7-NAF.DM. Excitation maximum for 2,7-NAF.DM is located near 500 nm. After excitation by UV radiation, the sample emits yellow–green light/glow, which coincides with the range of wavelength for green light (500–542 nm) and for the yellow one (542–578 nm) [23].

### Luminophor 2,7-NAF.DM Immobilization

Liquid-phase adsorption and chemisorption were applied for 2,7-NAF.DM immobilization on the surface of nanoparticles. Before modification with 2,7-NAF.DM, the silica (SiO_2_) and MWCNTs were activated by treatment with vinyl-containing trialkoxysilane (VTES). The modification of silica or carbon nanotubes was carried out in toluene solution of VTES with ethylenediamine as initiator of condensation of surface silanols (or phenolic groups on nanotube surface) with alkoxy groups. The process was stimulated by ultrasonic treatment and heating at 90 °C for 6 h in a closed reactor. Then, vinylated nanoparticles were dried at 110 °C until the complete solvent removal. As expected, the vinyl groups on the nanoparticles surface provide better affinity between the carriers and luminophor (2,7-NAF.DM) [24].

The modification process with 2,7-NAF.DM was carried out from toluene solution of monomer using azobisisobutyronitrile (AIBN) as attachment and polymerization initiator with heat and ultrasonic stimulation. In brief, demanded amount of the vinylated silica was dispersed in toluene solution of monomer by ultrasonic treatment (ultrasonic bath with frequency 44 kHz). After this, the dissolved initiator was introduced into dispersion and sonication was repeated. The composites with different carrier/monomer ratios were prepared (Table 1). Modification process was carried out at 80 °C for 24 h after sonication for 30 min. Then, samples were filtered, washed with toluene and acetone and dried at 110 °C until the complete solvent removal. The mechanical mixtures were prepared via milling of nanoparticles with cured solid polymer using Micro Ball Mill.

**Table 1:** Sample description

Sample	No.	Carrier mass, g	2,7-NAF.DM mass, g	AIBN mass, g	Immobilizing approach
Vinylated silica	1	1	–	–	–
Poly2,7-NAF.DM	2	–	1	0.1	–
SiO_2_ with polymer	3	0.2	0.1	–	Mechanical mixture
SiO_2_ with monomer polymerized	4	0.2	0.1	0.01	Chemisorption
MWCNTs with polymer in ratio 1:10	5	0.2	2	–	Mechanical mixture
MWCNTs with monomer polymerized in ratio 1:10	6	0.2	2	0.2	Chemisorption
MWCNTs with monomer polymerized in ratio 1:40	7	0.2	8	0.8	Chemisorption

The carrier/monomer ratio was established experimentally based on the reaction of the obtained materials on lighting of UV lamp. Assessment of the prospects of the samples was performed with a commercial 12-W UV lamp with radiation range 315–280 and 300–400 nm. For silica (white powder) that was used as a test sample, a luminescence under UV radiation is observed at smaller monomer (polymer) content in comparison with nanotubes (black powder), where luminescence was detected at higher concentrations of 2,7-NAF.DM.

#### Characterization of the Samples

The structure of the grafted surface layer was studied by means of thermal (differential scanning calorimetry (DSC), TGA) and spectral analysis. DSC thermograms were obtained using a DSC NETZSCH 204 calorimeter (NETZSCH, Günzbung, Germany). All thermal scans were carried out in aluminium pans with a sample weight of ~5–10 mg under nitrogen atmosphere (30 mL/min). The scans were performed at a heating rate of 10 °C/min in the temperature range of 20–500 °C.

Thermogravimetric (TGA) and differential thermal analysis (DTA) were performed with a Q-1500 D (MOM, Hungary) derivatograph. The measurements were made in corundum crucibles. The average sample mass was 100.6 ± 0.6 mg. The heating rate was 10 °C/min. The parameters of the derivatograph used were as follows: DTA 100 μV; TG 500 μV; differential thermo gravimetric (DTG) 500 μV; and sensitivity 20 or 50 mg. The pristine and modified with VTES MWCNTs were characterized using the thermal analyser STA 449F1 Jupiter (NETZSCH, Selb, Germany) with simultaneous TG-DSC analysis. All measurements were carried out in Al_2_O_3_ pans with a pierced lid with the sample mass 10 mg under artificial air atmosphere (20 mL min^−1^). Dynamic scans were performed at the heating rate of 10 K min^−1^ in the temperature range 30–1000 °C. Based on DSC and TGA results, the decomposition temperatures, the final decomposition mass, final decomposition temperature and enthalpy of process (Δ*H*_*d*_) were evaluated.

Room temperature UV–Vis absorption spectra were recorded using a V-660 JASCO spectrophotometer, and emission spectra were obtained with a Photon Technology International spectrofluorimeter equipped with a continuous wave xenon arc (Xe-arc) lamp as a light source. Spectral resolution was maintained at 1 nm. Liquid samples were measured in a quartz cuvette with a 2-mm optical path and solid samples—in appropriate adapters. UV–Vis reflectance spectra were registered using a horizontal sampling integrating sphere (Model PIV-756) connected to the V-660 spectrophotometer. The decay curves of polymer samples were excited at 450 nm by an Nd:YAG pulse laser (Opolette System, Quantel, USA) connected to the Photon Technology International spectrofluorimeter. Morphology of samples was studied by transmission electron microscopy using the Tecnai G2 20X-TWIN USA microscope.

## Results and Discussions

### Thermal Analysis of Nanocomposites Based on Silica

**Fig. 1:**
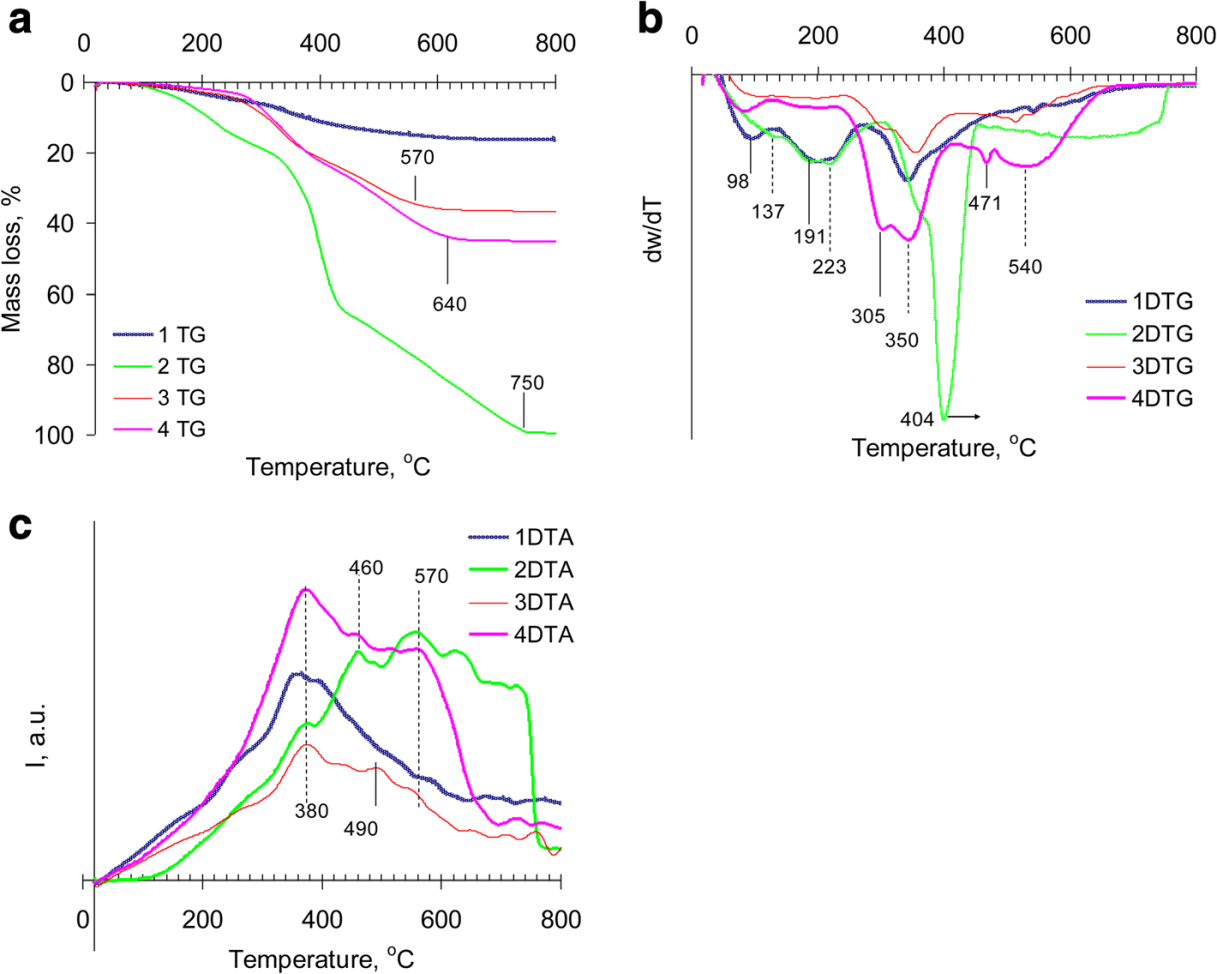
Thermal stability of raw materials and composites. **a** Mass loss curves. **b** Differential mass loss curves. **c** DTA curves. *1* The silica modified with vinyl trialkoxysilane; *2* the polymer 2,7-NAF.DM; *3* the mechanical mixture of the vinylated silica and the poly2,7-NAF.DM with the silica/polymer ratio 2:1 (sample 3 in Table 1); *4* the silica with chemisorbed polymeric layer obtained via in situ grafted polymerization of monomer 2,7-NAF.DM on the vinylated silica surface. Polymerization was carried out in toluene solution of the monomer in the presence of the dispersed silica and was initiated by AIBN during sonication with following heating at 80 °C for 24 h. The silica/polymer ratio in the composite was 2:1 (sample 4 in Table 1)

The polymer thermal decomposition goes through three mass loss stages with the total weight loss of 99 % (Fig. 1a). The first stage at 90–150 °C corresponded to solvent removal; the second stage at 150–300 °C was attributed to decomposition of terminal groups and ungrafted monomer evaporation; and the stage at 300–450 °C is coursed by main chain and benzene ring decomposition (Fig. 1b) [25–27]. The modified VTES silica has the total mass loss of 16 %. Decomposition process consists of a residual solvent removal (98 °C), alkoxy group and terminal vinyl group thermal oxidative destruction (130–250 °C) and degradation of the attached organosilicon layer (250–350 °C) (Fig. 1a, b).

The composite obtained by the mechanical mixing of vinylated silica (sample 3, Table 1) and polymer prepared by radical polymerization in a block (sample 4, silica/polymer ratio 2:1) loses 36.4 % of weight during thermooxidation. The initial decomposition temperature (*T*_in_) was observed at 230 °C that is higher than for pure polymer. The thermooxidation goes via two main stages with extrema of the mass removal rate at 308, 360 °C and maximum at 520 °C that can be attributed to the elimination of the polymer (or its degradation products) from the silica surface. The stages of silica surface ethoxy and terminal vinyl group thermooxidation as well as degradation of the terminal methacrylic, ester or hydroxyl groups of polymer in the interval 100–250 °C were not detected. It can be caused by interaction of the functional groups on silica surface with polymer during heating that promotes more thermally stable organic–inorganic composition capable of carbonizing. For the silica with immobilized polymer obtained via in situ polymerization in toluene solution, the thermooxidation process took place in two stages. At a low-temperature stage (300–400 °C) on the DTG curve, there are two peaks of the mass loss with maxima at 305 and 350 °C corresponding to the weight loss due to thermooxidation of the terminal groups [25, 26] and condensed benzene rings during grafted polymer depolymerisation [28]. Unlike degradation of pure polymer, the mass loss peaks of the main stage of thermooxidation were shifted to the lower temperatures.

At the same time, a narrow peak at 471 °C and a broad peak ranging from 450 to 660 °C with extremum at 540 °C (attributed to oxidation of the polymer layer directly on the silica surface) were detected on the DTG thermogram of sample 4 (Fig. 1b). After immobilization on the silica surface (via mechanical milling or by chemisorption), the polymer thermooxidation was observed at the lowest temperatures (Fig. 1c) probably due to silica surface’s ability to promote a thermooxidative degradation process [25]. The heat of process (Δ*H*) above 460 °C is decreased, and polymer destruction is completed at the lower temperature.

**Fig. 2:**
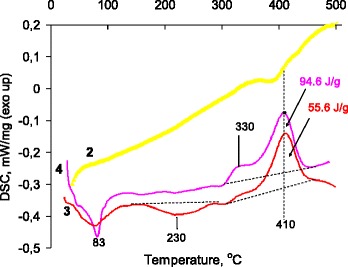
DSC analysis of the composites based on modified silica and 2,7-NAF.DM. This represents changes in the heat capacity of materials numbered as in Table 1. 3 The mechanical mixture of the vinylated silica with poly2,7-NAF.DM. The silica/polymer ratio was 2:1. The mechanical mixture of vinylated silica with polymer was obtained in a micro ball mill. *4* The silica with chemisorbed polymeric layer obtained via in situ grafted polymerization of the monomer 2,7-NAF.DM on the vinylated silica surface. The silica/polymer ratio in the composite was 2:1 (sample 4 in Table 1)

It should be noted that the degradation rates at the main decomposition stages (closely to 300 and 350 °C) for mechanically mixed and chemisorbed polymer (samples 3 and 4, Fig. 1b) differed, which indicates changes in composite polymeric structure. According to the DSC data (Fig. 2), the mechanically mixed with vinylated silica polymer after elimination of the volatile products at 83 °C has phase transition in the interval 150–270 °C with maximum on the endotherm at 230 °C corresponding to the melting process, which is not accompanied by a weight loss on the TG curve. In the range 300–460 °C on the DSC curve, exotherms with maxima at 330 and 410 °C were observed. These exotherms apparently correspond to chemical interaction of silica surface with products of the polymer thermal decomposition or post-curing of polymer into the surface layer. For chemisorbed polymer, the weak change in heat capacity at 210–230 °C (Fig. 1c, sample 4) can be attributed to glass transition and it can testify an amorphous structure of the attached layer. Also for sample 4, the heat of exothermal process is larger for mechanically mixed polymer, especially at 330 °C.

#### Thermal Analysis of Nanocomposites Based on Multiwalled Carbon Nanotubes

**Fig. 3:**
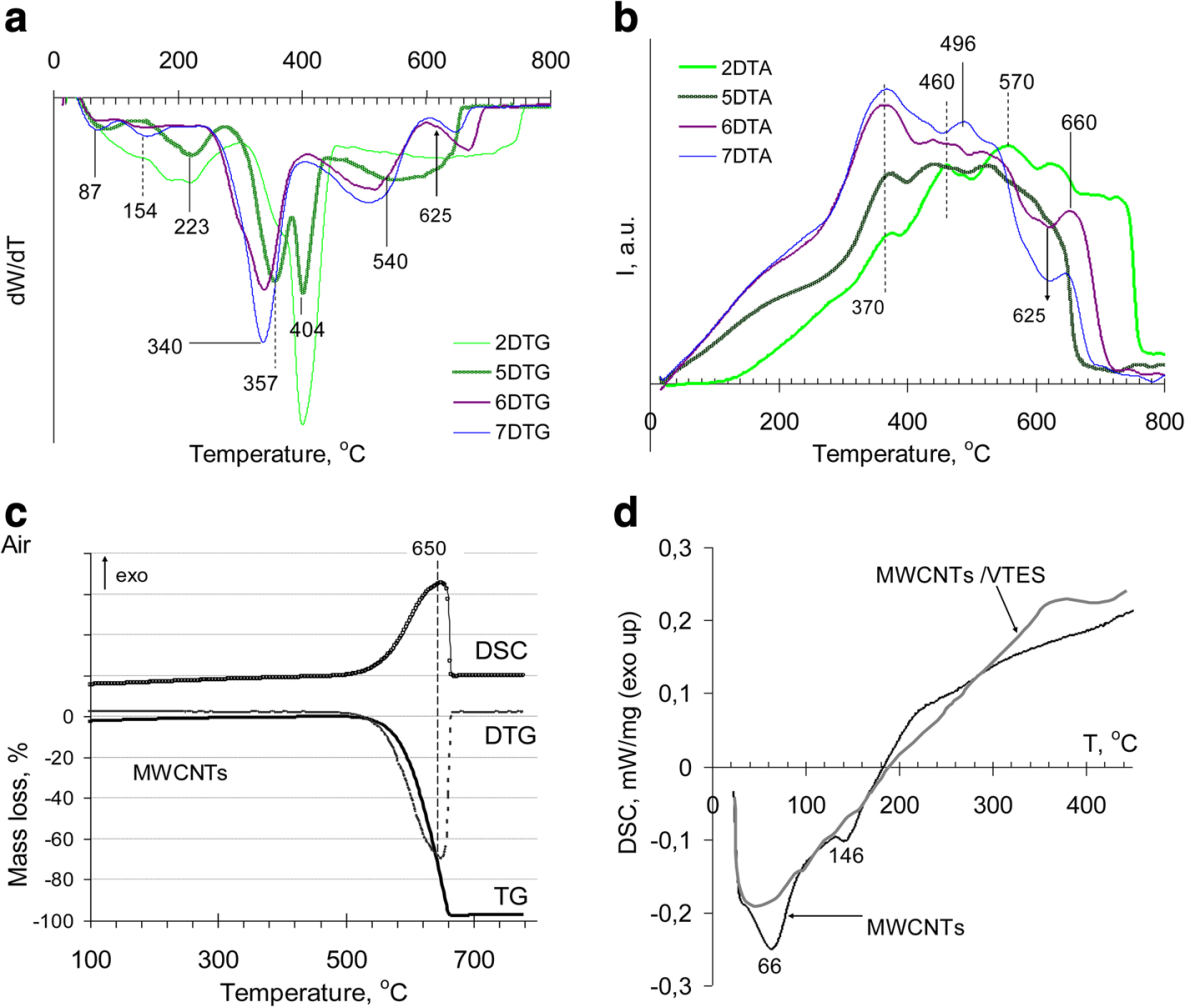
Thermal stability of raw materials and composites. **a** Differential mass loss curves (DTG). **b** DTA curves. **c** TG, DTG and DSC curves (artificial air). **d** DSC curves (nitrogen). The curves are marked as materials represented in Table 1. *2* The polymer 2,7-NAF.DM; *5* the mechanical mixture of carbon nanotubes with the poly2,7-NAF.DM. The MWCNTs/polymer ratio was 1:10; *6* carbon nanotubes with the polymeric layer obtained via in situ grafted polymerization of the monomer 2,7-NAF.DM. The MWCNTs/polymer ratio was 1:10; *7* carbon nanotubes with the polymeric layer obtained via in situ grafted polymerization of the monomer 2,7-NAF.DM. The MWCNTs/polymer ratio was 1:40; *black line* indicates the neat MWCNTs **(c, d)**; *grey line* indicates the vinylated MWCNTs **(d)** that were obtained by liquid-phase modification with the vinyl trialkoxysilane

The thermooxidation process of polymer mechanically milled with modified VTES carbon nanotubes (Table 1, sample 5) occurs in three stages with maxima of weight loss rates at 223, 340 and 404 °C with total mass loss of 96 % (Fig. 3a). It should be noted that for oxidized nanotubes, the starting decomposition temperature is 470 °C and the total mass loss is 99 % [29]. After nanotubes modification with silanes, the total mass loss was close to 96–98 % (Fig. 3c). A low content of a residual pitch after thermooxidation of modified with silane nanotubes was due to relatively low temperature of siloxane chains and attached silane oligomer degradation that occurs at temperatures below the nanotubes’ structural benzene ring destruction (closely to 250–320 °C) [30].

The splitting of main degradation peak testifies two kinds of polymer states, namely without and with interaction with the nanotube boundary layer. For composites with nanotube/polymer ratios 1:10 (sample 6) and 1:40 (sample *7*), the strong changes in thermooxidation process were not observed (Fig. 3a). But, the unlike silica-based composite, destruction of samples 6 and 7 (Table 1) in the range 250–410 °C occurs in one step with a maximum weight loss at 340 °C. This may indicate uniformity of the grafted layer. The different character of thermooxidation curves of mechanically mixed and chemisorbed polymer above 400 °C (Fig. 3a, curves 5 and 6) indicates the different nature of the polymer/carrier interaction, which leads to the formation of two types of thermooxidation products that decompose at 511 and 673 °C. For the pure polymer, there are no significant changes in the rate of mass loss in this temperature range (Fig. 3a, curve 2). But exotherms at 570 and 630 °C were noted (Fig. 3b, curve 2), whereas for the chemisorbed polymer, the exotherms were observed at about 520 and 660 °C. These data suggest that unlike polymer chemisorbed on silica, not only the interaction with grafted vinyl groups is realized in the surface layer of nanotubes but the orientation of condensed benzene rings of the polymer in the plane of the structural benzene rings of nanotubes is occurred [31, 32].

**Fig. 4:**
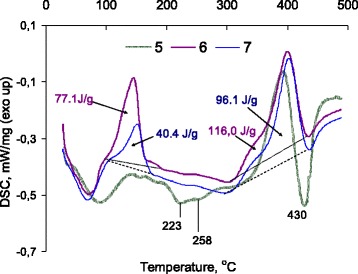
DSC analysis of the composites based on modified multiwalled carbon nanotubes and 2,7-NAF.DM. *5* Carbon nanotubes mechanically mixed with the poly2,7-NAF.DM with the MWCNTs/polymer ratio 1:10; *6* carbon nanotubes with the polymeric layer obtained via in situ grafted polymerization of the monomer 2,7-NAF.DM with the MWCNTs/polymer ratio 1:10; *7* carbon nanotubes with the polymeric layer obtained via in situ grafted polymerization of the monomer 2,7-NAF.DM with the MWCNTs/polymer ratio 1:40

As well as for the mechanical blend of the polymer with silica (Fig. 2), at heating of carbon nanotubes mixed with the polymer in the region near 223 °C on the DSC curve, the endotherm is detected (Fig. 4, curve 5), but in this case, the process is followed by the weight loss on the TGA curve (Fig. 3, curve 5). However, the endothermic peak at 258 °C (Fig. 4, curve 5) can be attributed to the polymer melting. In the temperature region of the polymer depolymerization, the exotherm at 330 °C, earlier marked for the composite with silica (sample 3, Table 1), was not observed and the exothermal process at 403 °C corresponding to post-curing of polymer goes into endothermic decomposition reaction (Fig. 4, curve 5). The endotherm at 430 °C corresponds to benzene ring degradation. At the polymer chemisorption in the low-temperature region (Fig. 4, curves 6 and 7), appearance of exotherms on the DSC curves testifies the chemical interaction in the grafted layer. At temperatures above 300 °C, the character of the DSC curves of samples 6 and 7 is similar to that of composite 4 (polymer chemisorption on the silica surface), but the heat of the process is increased. With the grafted polymer content increasing (samples 6 and 7), a heat of the exothermic process at 100–200 °C decreased with extremum shift towards high temperatures (Fig. 4, curve 7), which may be due to compaction of the polymeric layer grafted to the surface.

**Fig. 5:**
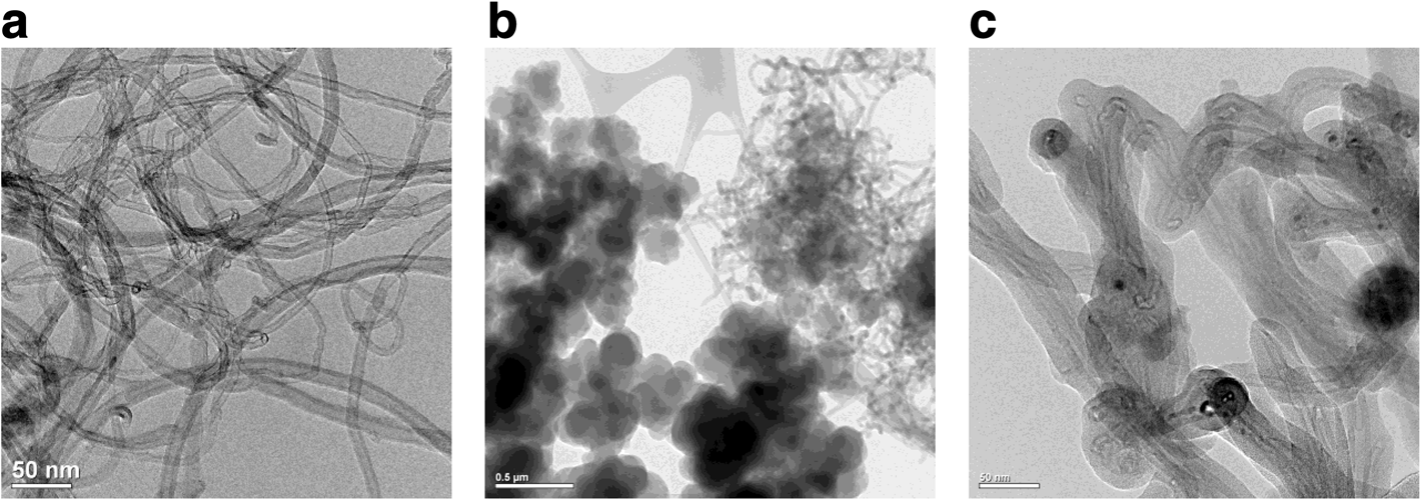
TEM images. **a** Pristine multiwalled carbon nanotubes (MWCNTs). **b** The mechanical mixture of carbon nanotubes with the poly2,7-NAF.DM. The MWCNTs/polymer ratio was 1:10 (sample 5 in Table 1). **c** Carbon nanotubes with chemisorbed layer of the poly2,7-NAF.DM. Chemisorption was realized via in situ grafted polymerization of the monomer on the nanotubes surface functionalized with vinyl trialkoxysilane. The MWCNTs/poly2,7-NAF.DM ratio was 1:40 (sample 7 in Table 1)

Implementation of chemisorption with simultaneous polymerization of the monomer on the surface of the vinylated nanotubes results in formation of homogeneous and amorphous polymeric layer. It was confirmed by TEM images (Fig. 5c). For mechanical mixture, nanotube/poly2,7-NAF.DM material contains a separated phase of polymer and carrier (Fig. 5b).

#### Photoluminescent Properties

**Fig. 6:**
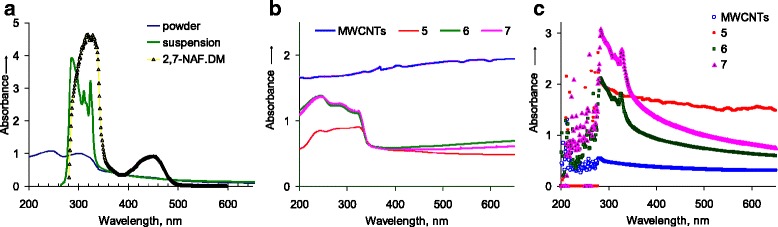
Absorption spectra. **a** The neat polymer (powder) and the silica with chemisorbed polymer 2,7-NAF.DM (dry powder and toluene dispersion). **b**, **c** Powder and toluene dispersion of carbon nanotubes (MWCNTs); *5* carbon nanotubes mechanically mixed with poly2,7-NAF.DM in ratio 1:10; *6* carbon nanotubes with the poly2,7-NAF.DM chemisorbed via in situ grafted polymerization with the MWCNTs/polymer ratio 1:10; *7* carbon nanotubes with the poly2,7-NAF.DM chemisorbed via in situ grafted polymerization with the MWCNTs/polymer ratio 1:40 (Table 1)

UV–Vis absorption spectra of the materials under this study in their powder form and dispersed in toluene are given in Fig. 6. The strong absorption in the UV region, up to 350 nm, obviously originates from naphthalene [21, 23]. The 450-nm band observed for pure polymer [23] and probably originates from the *π* conjugation in the naphthalene-diepoxymethacrylate moiety was not detected for 2,7-NAF.DM chemisorbed on the silica surface (Fig. 6). The 250-nm peak can be attributed to saturated compounds containing atoms with lone pairs (non-bonding electrons) capable of *n* → σ* transitions. Also, the aromatic residues (mostly phenyl) show bands near 250 nm [33]. Isomerization of polymer can result in 250-nm peak appearances too [34].

For composites based on carbon nanotubes, the presence of the bands at 240, 280 and 322 nm in the spectrum is characteristic, which are more evident in the spectra of dispersions (Fig. 6). Investigation of powders showed that only for chemisorbed polymer the strong bands, namely at 312 and 327 nm, were observed in the spectrum (Fig. 6, spectra 6 and 7).

**Fig. 7:**
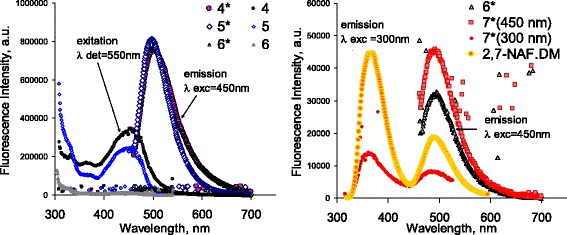
Excitation and emission spectra. This shows excitation (*) and emission spectra of the neat polymer (2,7-NAF.DM, *λ*_exc_ = 300 nm) and samples: 4* and *4* the silica with chemisorbed 2,7-NAF.DM; 5* and *5* carbon nanotubes mechanically mixed with the poly2,7-NAF.DM with the MWCNTs/polymer ratio 1:10; 6* and *6* carbon nanotubes with the poly2,7-NAF.DM chemisorbed via in situ grafted polymerization with the MWCNTs/polymer ratio 1:10 (spectra measured at *λ*_exc_ of 450 nm and *λ*_det_ of 550 nm); 7* carbon nanotubes with the poly2,7-NAF.DM chemisorbed via in situ grafted polymerization with the MWCNTs/polymer ratio 1:40 (spectra measured at *λ*_exc_ of 450 or 300 nm)

It has been found that the polymer chemisorption on the modified silica does not worsen its luminescent properties (Fig. 7, curve 4), whereas chemisorption on the modified nanotubes significantly reduces the intensity of excitation and emission bands at wavelengths characteristic for the pure polymer. Mechanical mixing of nanotubes with cured polymer has little effect on its luminescence but does not provide a sufficient composite’s uniformity.

Low intensity of the excitation and emission spectra of the polymer chemisorbed on carbon nanotubes is apparently attributable to a significant influence of the nanotube surface on the mechanism of electron transitions. The nature of surface interactions and conformation of grafted macromolecules are also important. Consequently, to achieve the luminescence intensity, it is necessary to hold the obtained degree of uniformity of the grafted polymer layer and ensure a certain orientation of polymeric macromolecules in the surface layer. It is known that increased interaction between the polymer molecules reduces the intensity of luminescence [23]; at the same time, it is not clear how the nanotube surface interact with the luminophor molecules. This aspect, as well as study of conditions that allow achieving positive effect of nanotubes surface on light intensity, demands more detailed investigation that will be carried out soon.

## Conclusions

The methods have been elaborated for luminophor chemisorption on fumed silica and MWCNTs by in situ attaching of the monomer via surface functional groups with simultaneous polymerization of 2,7-NAF.DM. Obtained results confirm the chemisorption of luminophor on the nanotubes and silica nanoparticles. After chemisorption the microstructure of 2,7-NAF.DM molecules is not changed, as opposed to the mechanical mixture. It was shown that the presented method allows one to obtain a homogeneous amorphous polymeric layer on the surface of nanoparticles. The polymer chemisorbed on the silica surface was found to retain its fluorescent properties (emission intensity neat and chemisorbed 2,7-NAF.DM was 3 × 10^6^ and 2.7 × 10^6^ a.u., respectively). It was noted that the polymer chemisorbed on MWCNTs has low intensity luminescence and no significant excitation in the wavelength range characteristic of pure polymer due to the peculiarities of orientation of the macromolecules in the surface layer of the carrier. The findings are the basis for optimizing the approaches to polymeric luminophor immobilization to achieve the desired structure of the graft polymer layer in order to improve the luminescence properties of composites based on carbon nanotubes or other carbon nanomaterials.

## Abbreviations

2,7.NAF: naphthalene-2,7-diol
2,7-NAF.DM: 2,7-(2-hydroxy-3-methacryloyloxypropoxy)naphthalene
AIBN: azobisisobutyronitrile
ATRP: atom transfer radical polymerization
DSC: differential scanning calorimetry
DTA: differential thermal analysis
DTG curve: differential thermo gravimetric curve
EP: epichlorohydrin
MA: methacrylic acid
MWCNTs: multiwalled carbon nanotubes
PMMA: poly(methyl methacrylate)
PMMA-b-PHEMA: poly(methyl methacrylate)-block-poly(hydroxyethylmethacrylate)
TGA: thermogravimetric analysis
UV: ultraviolet radiation
UV–Vis absorption: ultraviolet–visible absorption
VTES: vinyl trialkoxysilane
Δ*H*_*d*_: enthalpy of process
